# Group I metabotropic glutamate receptors differentially modulate excitatory transmission across interneuron types in the human cortex

**DOI:** 10.3389/fnsyn.2026.1766413

**Published:** 2026-02-13

**Authors:** Joanna Grace Sandle, Gábor Molnár, Martin Tóth, Katalin Ágnes Kocsis, Éva Adrienn Csajbók, Pál Barzó, Karri Lamsa, Gábor Tamás

**Affiliations:** 1 HUN-REN-SZTE Research Group for Cortical Microcircuits, Department of Physiology, Anatomy and Neuroscience, University of Szeged, Szeged, Hungary; 2 Doctoral School of Biology, University of Szeged, Szeged, Hungary; 3 Department of Neurosurgery, University of Szeged, Szeged, Hungary; 4 Hungarian Centre of Excellence for Molecular Medicine Research Group for Human Neuron Physiology and Therapy, Szeged, Hungary; 5 Research Group for Inhibitory Interneurons and Plasticity, Department of Physiology, Anatomy and Neuroscience, University of Szeged, Szeged, Hungary

**Keywords:** brain slice electrophysiology, fast-spiking interneurons, human neocortex, interneurons, synaptic modulation, type I metabotropic glutamate receptors

## Abstract

**Introduction:**

Group I metabotropic glutamate receptors (mGluRs) play a critical role in regulating neuronal excitability, synaptic strength, and cortical network activity. Although their physiological functions and involvement in neurological disorders are well established, direct experimental evidence for their role in human cortical neurons remains limited.

**Methods:**

We investigated the effects of group I mGluR activation on excitatory synaptic transmission in the human supragranular cortex using paired whole-cell patch-clamp recordings from synaptically connected pyramidal cells and interneurons in acute slices of human neocortex resected during neurosurgery.

**Results:**

Activation of mGluRs with the agonist (S)-3,5-dihydroxyphenylglycine (DHPG) altered excitatory synaptic efficacy in an interneuron subtype–dependent manner. Specifically, we observed acute enhancement of excitatory postsynaptic current (EPSC) amplitudes in 54% of fast-spiking interneurons and in 15% of non-fast-spiking interneuron types. Applying the same experimental protocol in slices from Wistar rats resulted in a similar increase in synaptic strength in fast-spiking interneurons. However, paired-pulse ratio analysis showed species-dependent differences, which may reflect distinct contributions of pre- and postsynaptic factors to the observed modulation.

**Discussion:**

Together, these results demonstrate that acute modulation of pyramidal cell–fast-spiking interneuron synapses via group I mGluRs is conserved between human and rodent neocortex, while pointing to species-specific underlying mechanisms. Moreover, mGluR-mediated modulation exhibits cell-type specificity in human cortical circuits. Collectively, these findings provide direct functional evidence for group I mGluR-dependent synaptic regulation in the human cortex and highlight important species- and cell-type–specific differences that should be considered when extrapolating rodent data to human cortical physiology and disease mechanisms.

## 1 Introduction

Cortical circuits are composed of excitatory and inhibitory synaptic connections formed by morphologically and functionally diverse neuronal populations, each playing distinct roles in shaping network dynamics, gain control, and temporal precision (Markram et al., 2004). The complexity of cortical processing arises from the heterogeneity of neuronal elements, the diversity of synaptic connectivity, and the ability of synapses to undergo activity-dependent changes in strength across multiple time scales (Lynch, 2004; Caporale and Dan, 2008; Nelson and Turrigiano, 2008; Collingridge et al., 2010). Glutamatergic excitatory transmission is finely regulated by metabotropic glutamate receptors (mGluRs), which modulate synaptic efficacy and neuronal excitability through intracellular signaling cascades (Pinheiro and Mulle, 2008; Sherman, 2014).

Among the three mGluR subgroups, group I mGluRs, comprising metabotropic glutamate receptor 1 (mGluR1) and metabotropic glutamate receptor 5 (mGluR5), are prominently involved in synaptic modulation and plasticity (Collingridge et al., 2010). These receptors are widely expressed in the rodent brain, including the cerebellar and cerebral cortices, and are preferentially localized to the perisynaptic region of postsynaptic densities at asymmetric synapses (Nusser et al., 1994; Luján et al., 1996). Their strategic subcellular localization positions them to sense glutamate spillover and regulate synaptic integration and plasticity.

The functional roles of group I mGluRs vary considerably across neuronal types and brain regions, contributing to the regulation of both excitatory and inhibitory signaling (Pinheiro and Mulle, 2008). In the neocortex and hippocampus these receptors are present both rodent and human (Tang et al., 2002; Boer et al., 2010; Szegedi et al., 2016; Kroon et al., 2019), but it is unknown whether modulation of specific neuron types by these receptors is similar in human and rodents. Group I mGluR effects are mediated largely through modulation of ion channels, including leak K^+^ channels, G-protein-coupled inwardly rectifying potassium (GIRK) channels, and voltage-gated N- and L-type Ca^2+^ channels (Anwyl, 1999). In the rodent cortex, group I mGluRs are critical for the induction of long-term potentiation (LTP) at glutamatergic synapses onto principal neurons (Vickery et al., 1997; Huemmeke et al., 2002; Wang and Daw, 2003; Stiefel et al., 2005; Wilson and Cox, 2007). In addition, LTP at excitatory synapses onto fast-spiking (FS) interneurons in layer 2/3 of the neocortex has been shown to depend on group I mGluR activation (Sarihi et al., 2008). Conversely, group I mGluRs have also been implicated in the induction of long-term depression (LTD) in the rodent visual cortex (Kato, 1993; Haruta et al., 1994; Hensch and Stryker, 1996) and in pyramidal cell-to-fast spiking interneuron synapses in human neocortex (Szegedi et al., 2016).

In the hippocampus, group I mGluR-dependent modulation and plasticity are well characterized and exhibit pronounced pathway- and target-specificity, particularly at interneuron synapses and distinct excitatory inputs (McBain et al., 1994; Hagena and Manahan-Vaughan, 2015; Lutzu et al., 2023). Similarly, in the rodent neocortex, group I mGluRs differentially regulate defined interneuron populations across somatosensory and visual cortical areas (Sarihi et al., 2008; Sun et al., 2009). In contrast, the modulatory roles and plasticity mechanisms of group I mGluRs in the human neocortex remain poorly understood. Among the limited available data, mGluR-dependent LTD has been demonstrated in fast-spiking interneurons receiving strong glutamatergic input from supragranular pyramidal cells (Szegedi et al., 2016). Furthermore, mGluR activation has been shown to depolarize human inhibitory interneurons, including Martinotti and fast-spiking basket cells (Kroon et al., 2019), suggesting a functional role in regulating human cortical excitability.

To better elucidate the mechanisms by which group I mGluRs modulate synaptic function in the human cortex, we aimed to characterize their effects on excitatory transmission onto interneurons by performing paired whole-cell recordings from synaptically connected neurons in both human and rat neocortical slices.

## 2 Results

### 2.1 Cluster analysis of firing properties defines fast-spiking and non-fast-spiking interneuron groups

In this study, we examined whether excitatory synapses onto GABAergic interneurons in the human cortex undergo modulation mediated by metabotropic glutamate receptor (mGluR) signaling pathways. Human cortical tissue was obtained from neurosurgical resections performed for the treatment of deep-brain tumors, hydrocephalus, and aneurysms. To investigate synaptic interactions, we performed paired whole-cell patch-clamp recordings from synaptically connected pyramidal cells and interneurons using a biocytin-containing intracellular solution, enabling *post hoc* morphological identification of a subset of recorded neurons.

**Figure 1:**
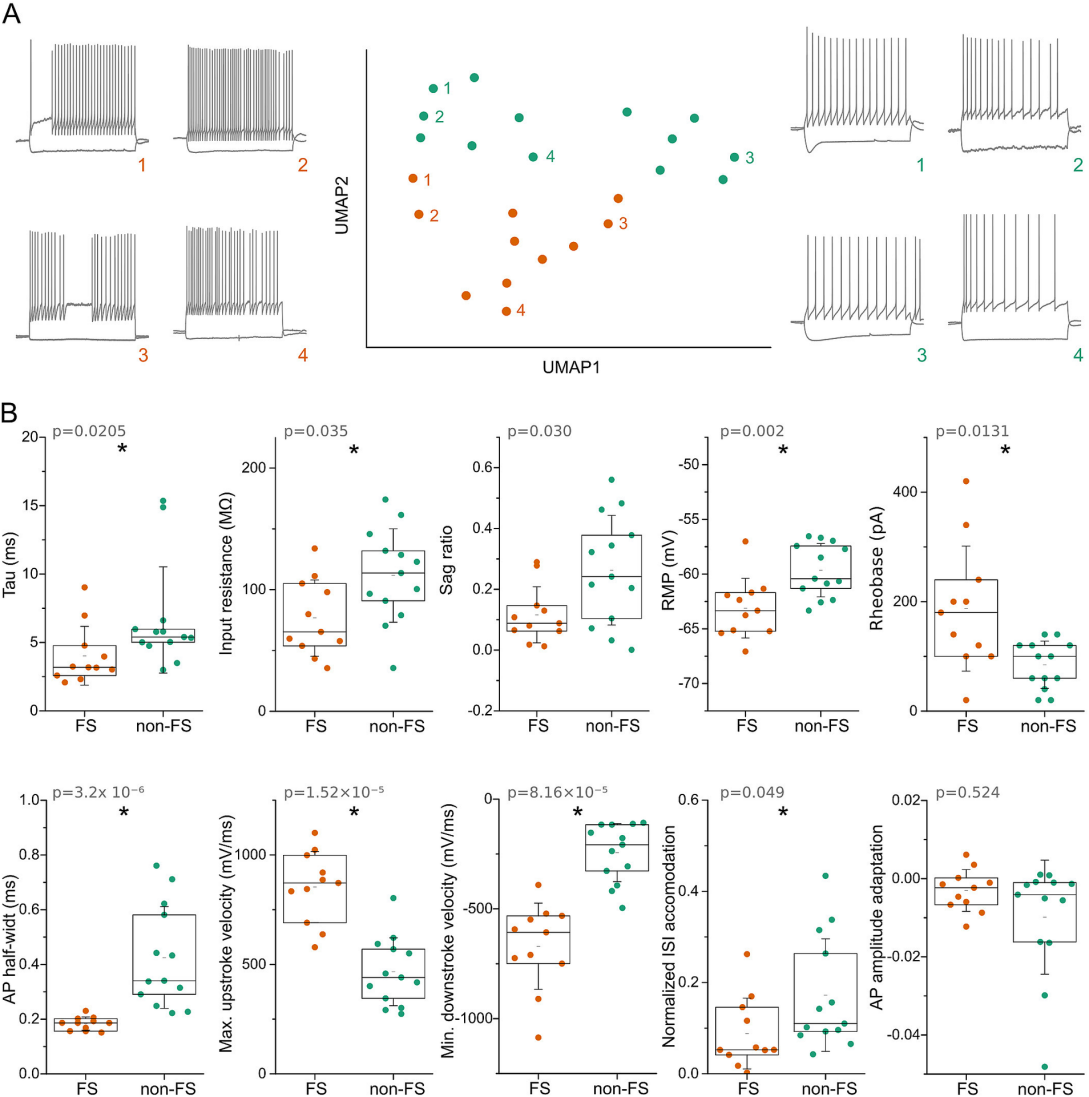
Electrophysiological classification of postsynaptic interneurons into fast-spiking and non-fast-spiking groups. **(A)** UMAP projection of interneurons based on electrophysiological features extracted from voltage responses to step current injections. Colors indicate the two clusters identified by *K*-means clustering: fast-spiking (FS, orange) and non-fast-spiking (non-FS, green). Representative membrane voltage responses to hyperpolarizing and depolarizing current steps are shown for FS (left) and non-FS (right) interneurons. **(B)** Comparison of intrinsic electrophysiological properties between FS and non-FS groups. Parameters include membrane time constant (*τ*), input resistance, sag ratio, resting membrane potential (RMP), rheobase, action potential (AP) half-width, maximum AP upstroke velocity, minimum AP downstroke velocity, normalized interspike interval (ISI) accommodation, and AP amplitude adaptation. Individual colored dots represent single neurons. Boxplots indicate the interquartile range (25th–75th percentile); the long black line denotes the median, the short black line the mean, and whiskers represent the standard deviation. Asterisks denote statistical significance (*p < 0.05).

Human GABAergic interneurons comprise multiple subtypes defined by distinct morphological, electrophysiological, and transcriptional properties (Chartrand et al., 2023; Lee et al., 2023). To characterize the diversity of postsynaptic interneurons in our dataset, we extracted 55 active and passive electrophysiological parameters from voltage responses to step current injections using custom-written MATLAB scripts. The resulting feature matrix was analyzed using Uniform Manifold Approximation and Projection (UMAP) for dimensionality reduction, followed by K-means clustering. This analysis revealed two electrophysiologically distinct groups of interneurons (Figure 1A).

The primary differences between these groups were related to action potential kinetics. We classified them as fast-spiking (FS) and non-fast-spiking (non-FS) interneurons. FS interneurons exhibited significantly shorter membrane time constants (*τ* = 4.03 ± 2.15 ms vs. 6.65 ± 3.88 ms, *p* = 0.0205, Mann–Whitney test), narrower action potential half-widths (0.18 ± 0.02 ms vs. 0.43 ± 0.19 ms, *p* = 3.2 × 10^−6^), and faster maximum rates of action potential rise and decay (upstroke: 853.01 ± 163.16 mV/ms vs. 466.63 ± 154.90 mV/ms, *p* = 1.52 × 10^−5^; downstroke: −670.27 ± 196.37 mV/ms vs. -243.87 ± 132.07 mV/ms, *p* = 8.16 × 10^−5^; Mann–Whitney tests for all comparisons; Figure 1B).

FS interneurons also displayed significantly smaller voltage sag responses to hyperpolarizing current injections (sag ratio: 0.161 ± 0.09 vs. 0.337 ± 0.20, *p* = 0.030) and a more hyperpolarized resting membrane potential compared with non-FS interneurons (−63.12 ± 2.72 mV vs. −59.64 ± 2.43 mV, *p* = 0.002; Mann–Whitney tests). Although input resistance varied across cells, FS interneurons had significantly lower mean input resistance (76.63 ± 31.37 MΩ vs. 111.60 ± 38.44 MΩ, *p* = 0.035). When normalized to spike count, FS interneurons showed significantly less interspike interval (ISI) accommodation than non-FS cells (normalized ISI accommodation: 0.088 ± 0.078 vs. 0.172 ± 0.123, *p* = 0.049). Action potential amplitude adaptation was minimal in both groups, although a subset of non-FS interneurons exhibited mild adaptation (−0.0030 ± 0.005 vs. -0.0099 ± 0.0146, *p* = 0.524; Figure 1B). Additionally, FS interneurons had significantly lower rheobase values (84.62 ± 43.32 pA vs. 187.27 ± 114.29 pA, *p* = 0.013) and larger action potential amplitudes (96.51 ± 9.45 mV vs. 79.25 ± 15.00 mV, *p* = 0.045).

The electrophysiological profile of the FS interneuron group, short membrane time constant, rapid action potential kinetics, and narrow spike width, corresponds closely to previously described fast-spiking interneurons in rodent (Kawaguchi and Kubota, 1997; Ascoli et al., 2008; Lee et al., 2023) and human (Szegedi et al., 2016, 2024; Wilbers et al., 2023). These properties are characteristic of parvalbumin-expressing (PV+) interneurons (Somogyi, 1977; Somogyi et al., 1983; Gulyás et al., 1993) and are most consistent with the small basket cell subtype (Markram et al., 2004).

Each recorded neuron was subjected to *post hoc* morphological analysis after complete staining and recovery (see Methods), allowing detailed examination of dendritic, axonal, and somatic structures (full anatomical recovery: n = 13 of 24 cells). Among fast-spiking interneurons, seven of 11 cells were successfully reconstructed. Of these, six were identified as basket cells, characterized by densely branched axons surrounding pyramidal cell somata or innervating perisomatic dendrites and multipolar dendritic trees (Tremblay et al., 2016), and one was identified as an axo-axonic (chandelier) cell. The axo-axonic cell displayed characteristic axonal cartridges targeting the axon initial segments of pyramidal neurons (Somogyi, 1977; Szabadics et al., 2006) and exhibited a firing pattern typical of human axo-axonic cells (Szabadics et al., 2006; Molnár et al., 2008), consistent with our previous observations, where suprathreshold depolarizing current steps (0.8 s) evoked a single spike or brief burst at stimulus onset, followed by a 100–300 ms pause and subsequent high-frequency firing (Figure 1A, top left).

To further validate interneuron identity, we performed immunohistochemical labeling for parvalbumin in a subset of recorded cells (Somogyi, 1977; Somogyi et al., 1983; Markram et al., 2004). However, due to the prolonged recording protocol, intracellular components were substantially diluted, rendering immunolabeling unreliable (Pusch and Neher, 1988). Consequently, we could not systematically confirm parvalbumin expression in the recorded neurons. A subset of fully filled cells with anatomic recovery (n = 6) revealed multipolar interneurons with approximately spherical axonal arborizations and thin dendrites, lacking morphological features characteristic of basket and axo-axonic cells; these were classified as non-FS interneurons. Two of these cells displayed neurogliaform morphology.

### 2.2 Group I mGluR activation differentially modulates excitatory synaptic strength onto layer 2/3 interneurons

#### 2.2.1 Diverse modulation of excitatory inputs to layer 2/3 fast-spiking interneurons by group I mGluRs in the human neocortex

**Figure 2:**
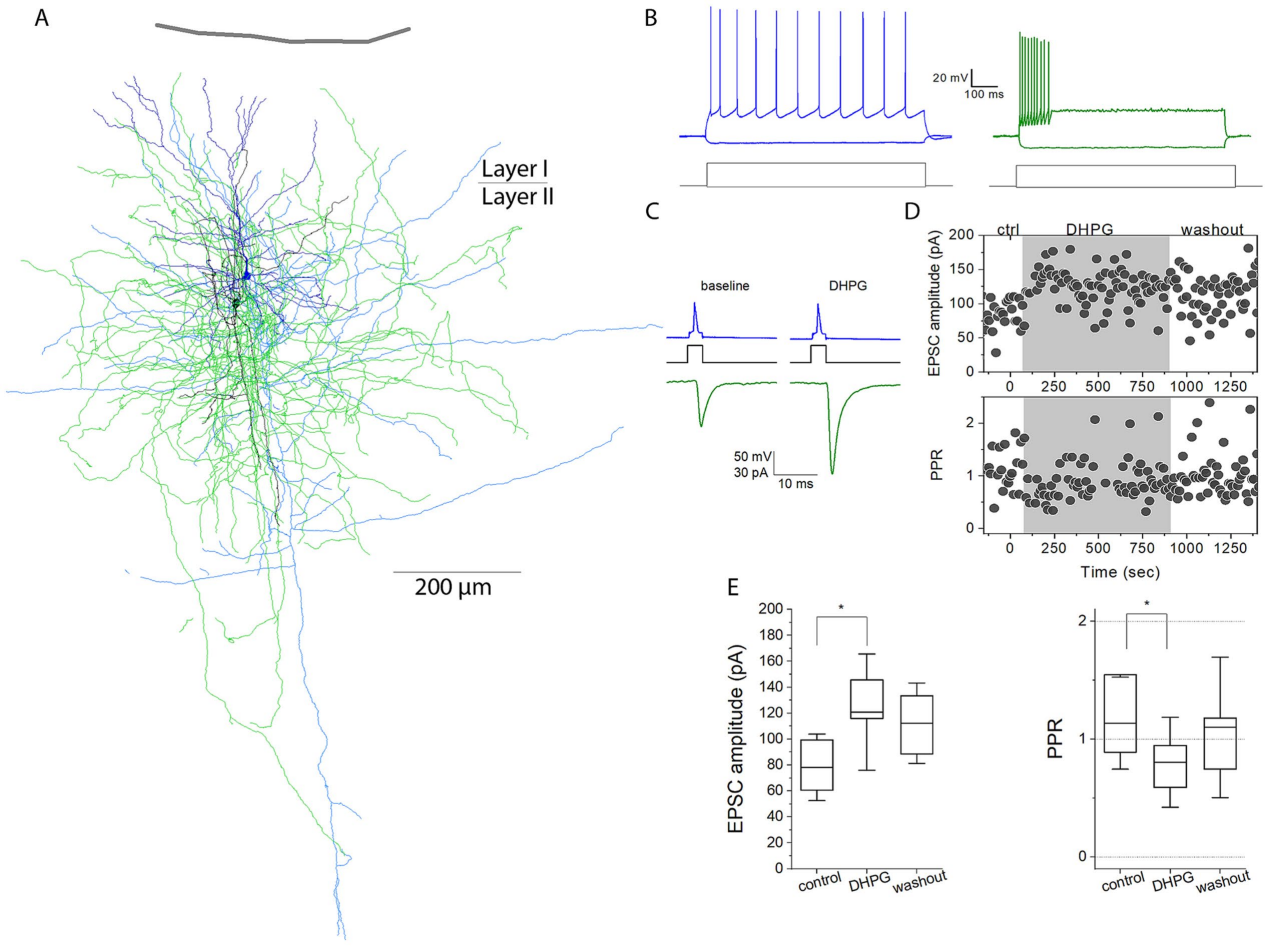
The effect of group I mGluR activation on monosynaptic excitation of layer 2/3 basket cells in the human cortex. **(A)** Light microscopic reconstruction of the recorded pyramidal cell (dendrites: dark blue; axon: light blue) and basket cell (dendrites: dark green; axon: light green). **(B)** Representative firing patterns of the simultaneously recorded presynaptic pyramidal neuron (left) and postsynaptic basket cell (right). **(C)** Action potentials in the presynaptic pyramidal neuron (top) evoked unitary EPSCs in the basket cell (bottom) under voltage-clamp conditions before (left) and after (right) application of the group I mGluR agonist DHPG. **(D)** Time course of EPSC amplitudes and PPR during the experiment. Bath application of DHPG started at time 0 and is indicated by the gray shaded background. **(E)** Left: DHPG induced a significant increase in EPSC amplitudes in this representative experiment (Mann–Whitney test). Boxes represent the mean and interquartile range (IQR) of EPSC amplitudes during baseline (−200 to 0 s) and after DHPG application (60–260 s). Right: Paired-pulse ratio (PPR) measured using two stimuli delivered at a 50 ms interval during baseline and following DHPG application. Whiskers indicate the standard deviation. Asterisks denote statistical significance (*p < 0.05).

To investigate how group I mGluRs modulate excitatory synaptic input to inhibitory interneurons, we recorded excitatory postsynaptic currents (EPSCs) evoked by paired-pulse stimulation in layer 2/3 pyramidal cell-interneuron pairs in acute human cortical slices (Figure 2). Interneurons were classified as fast-spiking (FS; n = 11) or non-fast-spiking (non-FS; n = 13) based on cluster analysis of electrophysiological features (Figure 1A). Functional monosynaptic connections were detected in approximately 15% of all tested pyramidal cell–FS and pyramidal cell-non-FS pairs. Paired-pulse stimulation alone did not alter synaptic strength over the recording period (n = 4; baseline EPSC amplitude: 81.57 ± 71.59 pA; after 6 min control: 81.67 ± 71.05 pA; *p* = 0.93, paired t-test), indicating stable baseline conditions.

**Figure 3:**
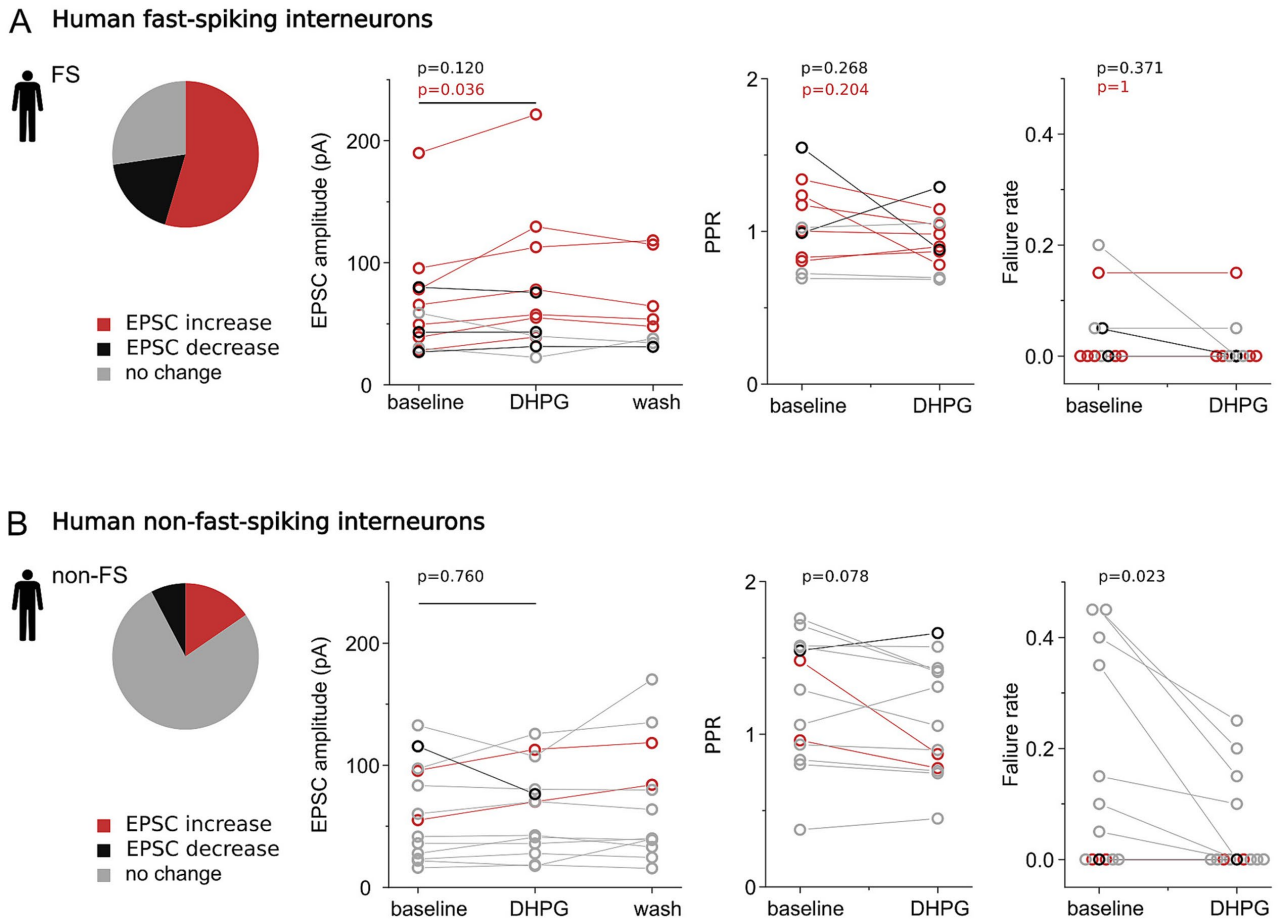
Group I mGluR–mediated modulation of excitatory monosynaptic inputs onto human fast-spiking and non-fast-spiking interneurons. **(A)** Fast-spiking interneurons. Pie chart depicts the proportion of synapses showing significant EPSC increase (red), decrease (black), or no significant change (light gray) following DHPG application. Line plots show the time course of average EPSC amplitudes during baseline, DHPG application, and washout, along with paired-pulse ratio (PPR) and holding currents measured before and after DHPG. **(B)** Non-fast-spiking interneurons. Same format as in **(A)**, summarizing experiments with non-FS interneurons as postsynaptic targets.

Baseline EPSC amplitudes in FS interneurons ranged from 26.88 to 189.84 pA (mean: 62.66 ± 46.24 pA), with a mean synaptic latency of 0.98 ± 0.21 ms and a paired-pulse ratio (PPR) of 1.03 ± 0.27 (second/first EPSC). Following baseline recording, the group I mGluR agonist (S)-3,5-dihydroxyphenylglycine (DHPG; 50 μM) was bath-applied, and synaptic responses were continuously monitored (Figure 2). DHPG induced heterogeneous changes in synaptic efficacy across FS interneurons. In 54% of recordings (n = 6), DHPG significantly increased EPSC amplitudes (baseline: 75.04 ± 59.05 pA; DHPG: 96.78 ± 68.63 pA; *p* = 0.036, Wilcoxon signed-rank test; Figure 3A). In contrast, two FS cells exhibited significant EPSC attenuation (baseline: 44.54 ± 20.26 pA; DHPG: 31.23 ± 12.35 pA), while EPSCs remained unchanged in the remaining three FS interneurons (baseline: 75.28 ± 55.27 pA; DHPG: 75.19 ± 53.48 pA). Consistent with this heterogeneity, no significant change was detected at the population level (baseline: 62.66 ± 46.24 pA; DHPG: 72.14 ± 57.62 pA; n = 11; *p* = 0.120, Wilcoxon signed-rank test).

In a subset of experiments, DHPG was washed out after 3–4 min by perfusion with agonist-free artificial cerebrospinal fluid (ACSF) identical to one used in baseline conditions. EPSC amplitudes in synapses that had been strengthened during DHPG application gradually returned toward baseline values during washout (n = 4; baseline: 58.07 ± 17.33 pA; DHPG: 79.10 ± 34.58 pA; washout 4 min: 70.22 ± 30.56 pA; washout 8 min: 62.97 ± 31.24 pA). In one experiment, DHPG was washed out with mGluR1 and mGluR5 antagonists LY367385 and MPEP, respectively. The observed reduction in synaptic strength was also reversed with washout (one with ACSF, one with antagonists) (n = 2, baseline: 44.54 ± 20.26 pA, DHPG: 31.23 ± 12.35 pA, washout for 4 min: 36.08 ± 2.45 pA, washout for 8 min: 38.22 ± 8.23 pA; Figure 3A).

Changes in synaptic strength can arise through either presynaptic or postsynaptic mechanisms, including altered neurotransmitter release probability or changes in postsynaptic AMPA receptor density and conductance. To distinguish between pre- and postsynaptic contributions, we examined paired-pulse ratios and failure rates. DHPG did not significantly alter PPR at pyramidal cell–FS interneuron synapses, either when analysis was restricted to synapses showing significant increases in EPSC amplitude (baseline: 1.08 ± 0.24; DHPG: 0.90 ± 0.17; n = 6; *p* = 0.204, Wilcoxon signed-rank test) or when all recorded FS interneurons were included (baseline: 1.03 ± 0.27; DHPG: 0.94 ± 0.19; n = 11; *p* = 0.268, paired t-test; Figure 3A). Similarly, synaptic failure rates remained low and were not significantly affected by DHPG (baseline: 0.041 ± 0.070; DHPG: 0.018 ± 0.046; *p* = 0.371, Wilcoxon signed-rank test) although failures were completely eliminated in two cases (baseline failure rate: 0.041 ± 0.070, DHPG failure rate: 0.018 ± 0.046, p = 0.371, Wilcoxon Signed Rank test; Figure 3A). Together, these findings indicate that mGluR-dependent synaptic enhancement in human FS interneurons most likely reflects a postsynaptic mechanism.

#### 2.2.2 Group I mGluR activation has modest effects on excitatory synaptic strength in non-fast-spiking interneurons

Thirteen interneurons receiving excitatory synaptic input were classified as non-fast-spiking (non-FS) based on their electrophysiological properties. This heterogeneous population exhibited slower action potential kinetics and more prominent voltage sag compared to FS interneurons, consistent with the classification. However, other electrophysiological features were variable, indicating the presence of multiple interneuron subtypes within this group. One notable source of heterogeneity was firing pattern adaptation: some interneurons fired regularly throughout depolarizing current steps, whereas others displayed pronounced spike-frequency adaptation.

Under baseline conditions, EPSC amplitudes in non-FS interneurons ranged from 15.97 to 132.64 pA (mean: 61.99 ± 38.97 pA), with an average latency of 1.23 ± 0.55 ms and a paired-pulse ratio (PPR) of 1.22 ± 0.42.

In contrast to FS interneurons, group I mGluR activation by DHPG produced only modest changes in synaptic strength in the non-FS group (*n* = 13; baseline: 61.99 ± 38.97 pA; DHPG: 63.53 ± 36.37 pA; *p* = 0.760, paired *t*-test), with most synapses remaining unchanged (*n* = 10; baseline: 53.98 ± 38.81 pA; DHPG: 56.66 ± 37.67 pA; *p* = 0.554; paired t-test; Figure 3B and Supplementary Figure 2). During washout, a slight further increase in EPSC amplitudes was observed (*n* = 10; washout at 4 min: 64.03 ± 50.85 pA; washout at 8 min: 59.15 ± 53.05 pA).

In two experiments, EPSC amplitudes were significantly increased to a degree comparable to that observed in FS interneurons. In both cases, the observed modulation persisted throughout the initial 4 min of washout and stabilized by 8 min (*n* = 2; baseline: 75.26 ± 28.59 pA; DHPG: 91.43 ± 30.16 pA; washout at 4 min: 101.15 ± 24.34 pA; washout at 8 min: 94.96 ± 34.21 pA; Figure 3B). Conversely, one non-FS interneuron exhibited significant synaptic attenuation following DHPG application (baseline: 115.47 ± 47.66 pA; DHPG: 76.36 ± 32.63 pA).

In the non-FS experiments showing EPSC enhancement, we observed a trend toward reduced PPR (*n* = 2, baseline: 1.22 ± 0.37, DHPG: 0.82 ± 0.06), whereas changes in PPR were more heterogeneous across all non-FS cells (*n* = 13, baseline: 1.22 ± 0.43, DHPG: 1.10 ± 0.38, *p* = 0.078, paired *t*-test). Human PC to non-FS interneuron synapses exhibited low failure rates, but higher than PC-FS connections. Here, DHPG application significantly reduced failure rates (baseline: 0.15 ± 0.19, DHPG: 0.054 ± 0.09, *p* = 0.022, Wilcoxon Signed Rank test).

#### 2.2.3 Excitatory inputs onto layer 2/3 basket and axo-axonic cells are modulated by mGluR activation in the rat cortex

To assess whether the mGluR-mediated modulation of synaptic strength observed in human interneurons is conserved across species, we applied the same experimental protocol in layer 2/3 of the somatosensory cortex of Wistar rats. Based on our human data, we focused on fast-spiking (FS) interneurons, which exhibited the most pronounced modulation by group I mGluR activation.

Rodent FS interneurons displayed electrophysiological properties similar to those of human FS interneurons, including a short membrane time constant (2.94 ± 0.77 ms), narrow action potential (AP) half-width (0.22 ± 0.08 ms), and rapid upstroke and downstroke velocities (upstroke: 603.88 ± 254.17 mV/ms; downstroke: −545.90 ± 279.92 mV/ms). However, several species-specific differences were evident: rodent FS neurons had a lower input resistance (49.95 ± 18.12 MΩ), higher rheobase current (377.14 ± 169.32 pA), and smaller AP amplitudes (77.90 ± 16.91 mV) (Bakos et al., 2025). Morphological reconstruction of recovered interneurons (*n* = 7 of 14) revealed basket cells (*n* = 6) and a single axo-axonic cell, based on characteristic axonal arborization patterns.

In control experiments without receptor agonists, synaptic strength remained stable over time in paired recordings (*n* = 6, baseline: 98.82 ± 124.53 pA; control period: 96.51 ± 110.61 pA; *p* = 1.0, Wilcoxon signed-rank test). When the group I mGluR agonist DHPG was applied (*n* = 14), baseline EPSC amplitudes ranged from 26.02 to 268.25 pA, with a mean of 95.66 ± 85.56 pA and a paired-pulse ratio (PPR) of 1.33 ± 0.59.

**Figure 4:**
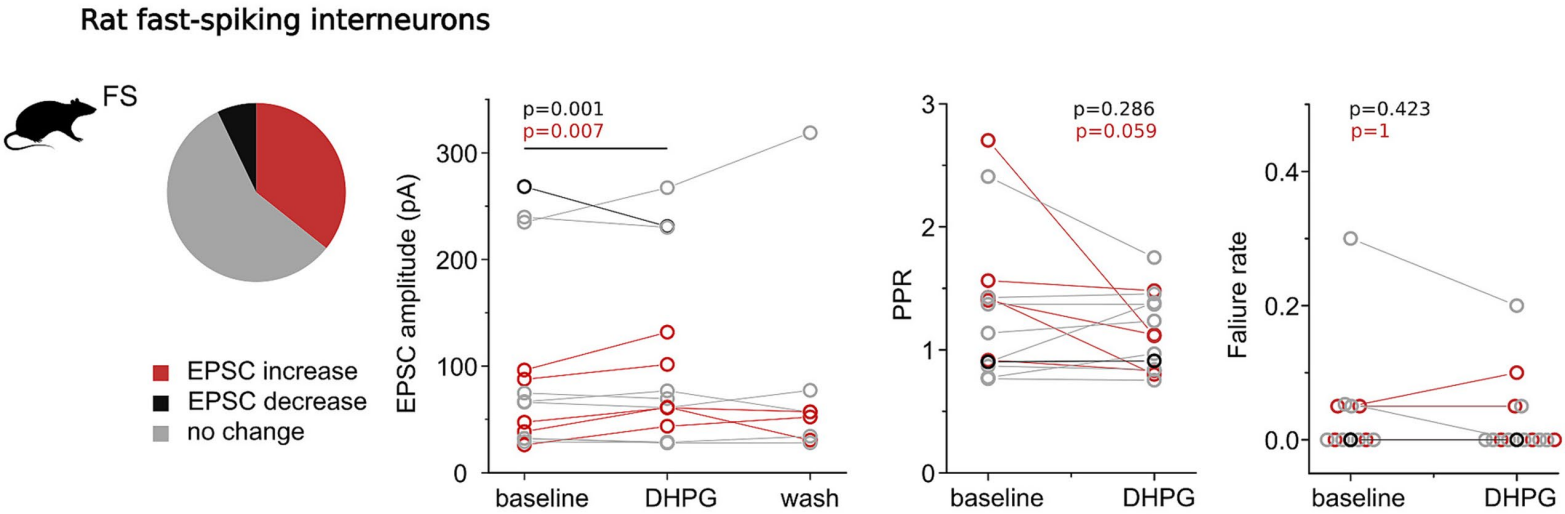
Group I mGluR–mediated modulation of excitatory monosynaptic inputs onto fast-spiking interneurons in rat neocortex. The pie chart shows the proportion of synapses exhibiting significant decrease (black), increase (red), or no significant change (light gray) in EPSC amplitude following DHPG application. Line plots show mean EPSC amplitudes for individual interneurons during baseline, DHPG application, and washout periods. Additional line plots depict paired-pulse ratios (PPR) and holding currents before and during DHPG application.

Overall, activation of group I mGluRs led to a significant increase in excitatory synaptic strength at FS interneurons (baseline: 95.67 ± 85.56 pA; DHPG: 101.39 ± 82.10 pA; *p* = 0.001, Wilcoxon signed-rank test). As in the human cortex, however, this effect was heterogeneous: 36% of connections showed significant increase in EPSC amplitude (*n* = 5; baseline: 59.17 ± 31.04 pA; DHPG: 79.83 ± 35.99 pA; *p* = 0.007, paired t-test), one connection exhibited significant decrease (baseline: 268.24 pA; DHPG: 230.82 pA), and most showed no significant change (*n* = 8; baseline: 96.90 ± 88.52 pA; DHPG: 98.68 ± 95.08 pA; Figure 4). One experiment showed a delayed but significant increase in EPSC amplitude approximately 3 min after DHPG application and was therefore grouped with the strengthening experiments.

In three positively modulated experiments, EPSC amplitudes recovered towards baseline following washout, indicating that the enhancement was dependent on continued receptor activation (*n* = 3; baseline: 37.30 ± 10.71 pA; DHPG: 55.30 ± 10.20 pA; washout: 47.62 ± 14.17 pA). Correspondingly, in the single attenuated case, EPSC amplitude recovered by increasing during washout.

In experiments showing DHPG-induced increase in EPSC amplitude, there was a trend toward a reduction in PPR that did not reach statistical significance (*n* = 5; baseline: 1.60 ± 0.66; DHPG: 1.08 ± 0.28; *p* = 0.059, Wilcoxon signed-rank test). By contrast, PPR showed no consistent change in experiments with reduced or unchanged EPSC amplitudes, nor when all experiments were pooled (*n* = 11; baseline: 1.33 ± 0.59; DHPG: 1.14 ± 0.31; *p* = 0.286, Wilcoxon signed-rank test).

Failure rates at rodent FS synapses were generally low and were not significantly affected by DHPG (all connections: *n* = 14; baseline: 0.036 ± 0.080; DHPG: 0.021 ± 0.054; *p* = 0.181; strengthening connections: *n* = 5; baseline: 0.020 ± 0.027; DHPG: 0.030 ± 0.045; *p* = 1.0; Wilcoxon signed-rank test), consistent with a predominantly postsynaptic mechanism of modulation.

#### 2.2.4 Baseline synaptic properties differentially predict mGluR-induced modulation at human and rodent PC–FS synapses

The heterogeneity of synaptic responses to group I mGluR activation in fast-spiking (FS) interneurons prompted us to examine whether baseline synaptic or presynaptic parameters could predict the direction and magnitude of EPSC amplitude modulation. Previous studies have suggested that presynaptic release probability, membrane potential and Ca^2+^-dependent vesicle recruitment can influence both the direction and magnitude of synaptic plasticity at excitatory synapses (Larkman et al., 1992; Trommershäuser et al., 2003; Körber and Kuner, 2016; Böhme et al., 2018; Monday et al., 2018).

**Figure 5:**
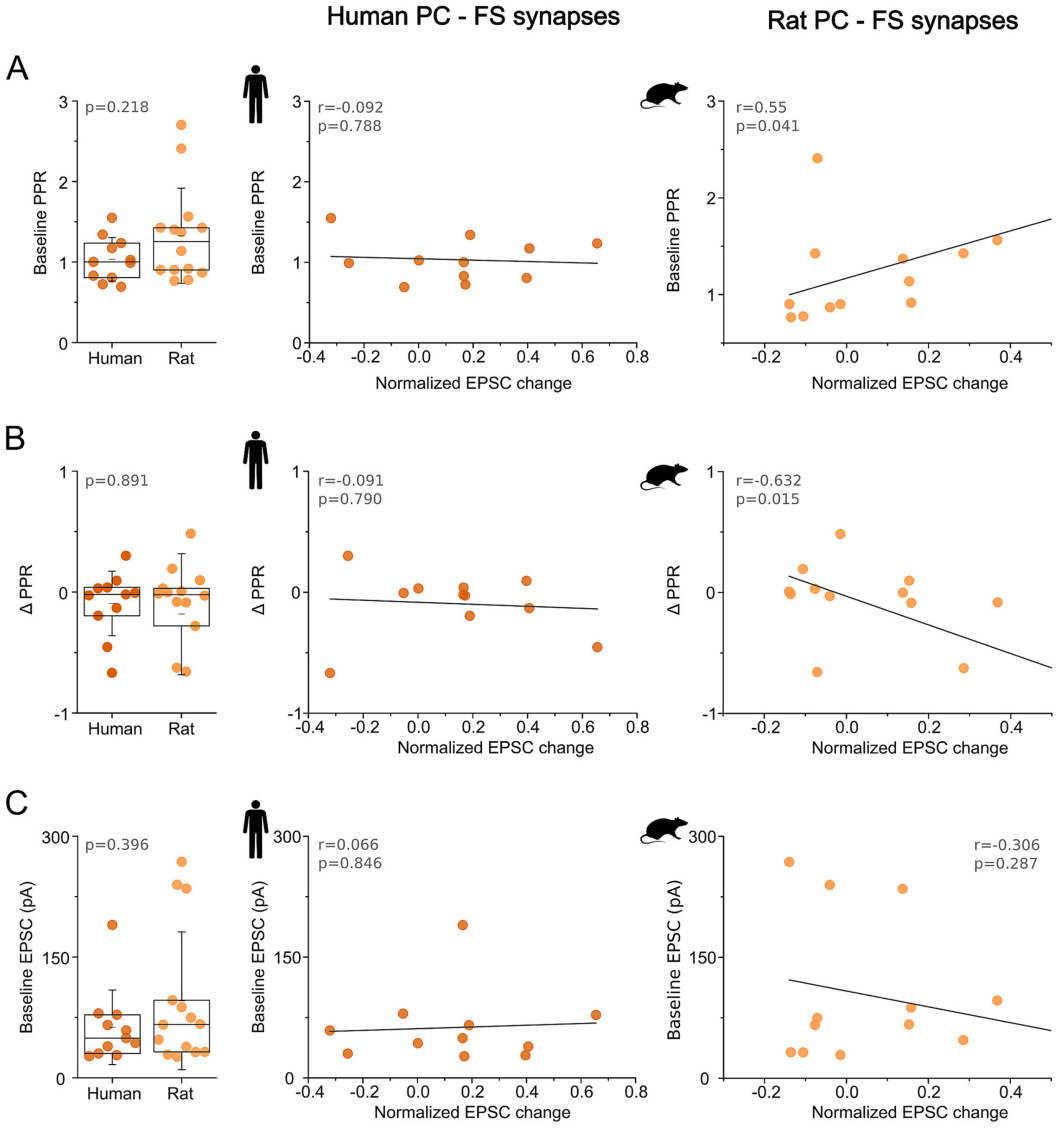
Baseline synaptic properties predict mGluR-induced modulation differently in human and rat PC–FS synapses. **(A)** Box plots show baseline paired-pulse ratio (PPR) values for human and rat pyramidal cell (PC) to fast-spiking (FS) interneuron connections. Each dot represents an individual monosynaptically connected PC–FS pair. Boxes indicate the interquartile range (25th–75th percentiles), the horizontal line marks the median, the short black line indicates the mean, and whiskers denote the standard deviation. The middle scatter plot shows the linear regression between baseline PPR and the normalized change in EPSC amplitude following group I mGluR activation in human FS interneurons. The right scatter plot shows the corresponding analysis for rat PC–FS connections. Solid lines indicate best-fit linear regressions. **(B)** Same as in panel **(A)**, showing DHPG-induced change in PPR in human and rat PC-FS pairs. **(C)** Same format as **(A,B)** but plotted against baseline EPSC amplitude instead of PPR.

To quantify synaptic changes, we normalized the difference between mean EPSC amplitudes during DHPG application and baseline recordings to the baseline amplitude. Pearson correlation analysis revealed no association between normalized synaptic change and baseline paired-pulse ratio (PPR) in human synapses (*n* = 11, *r* = −0.092, *p* = 0.788). In contrast, synapses exhibiting higher initial PPR in the rat cortex tended to show an enhancement in synaptic strength, as evidenced by a significant positive correlation (*n* = 14, *r* = 0.552, *p* = 0.040), indicating that rodent synapses with lower initial release probability were more likely to undergo EPSC enhancement following group I mGluR activation (Figure 5A).

Moreover, at rat FS synapses, ΔPPR (mean PPR during DHPG minus mean PPR measured under baseline recordings) was significantly negatively correlated with the normalized EPSC change (*n* = 14, *r* = −0.632, *p* = 0.015), such that synapses showing stronger DHPG-induced enhancement also exhibited larger decreases in PPR. While, in human synapses ΔPPR did not correlate with the magnitude of EPSC change (*n* = 11, *r* = −0.091, *p* = 0.790; Figure 5B). This coupling between EPSC increase and a change in PPR is consistent with a DHPG-evoked increase in presynaptic release probability at rat PC–FS synapses and suggests that, in rodents, group I mGluR-mediated modulation of synaptic strength are expressed predominantly through presynaptic mechanisms.

We next examined whether baseline synaptic strength predicted the direction and magnitude of the DHPG-induced modulation. However, no significant relationship was detected between initial EPSC amplitude and normalized synaptic change in either species (human: *n* = 11, *r* = 0.066, *p* = 0.846; rat: *n* = 14, *r* = −0.306, *p* = 0.287), indicating that baseline synaptic strength did not account for the observed differences in the expressed modulation (Figure 5C).

### 2.3 Group I metabotropic glutamate receptors alter the membrane potential and intrinsic properties of interneurons

Group I mGluRs are known to modulate multiple ion channel types, predominantly affecting K^+^ conductances (Chemin, 2003). Such modulation can alter membrane potential and intrinsic excitability, thereby influencing the induction and expression of synaptic plasticity, including spike-timing-dependent plasticity (STDP), LTP, and short-term plasticity (Fernández-Fernández and Lamas, 2021). To determine whether the synaptic effects observed in this study were associated with changes in intrinsic membrane properties, we analyzed holding current and input resistance before and after application of the group I mGluR agonist DHPG.

#### 2.3.1 Effects in human cortical interneurons

**Figure 6:**
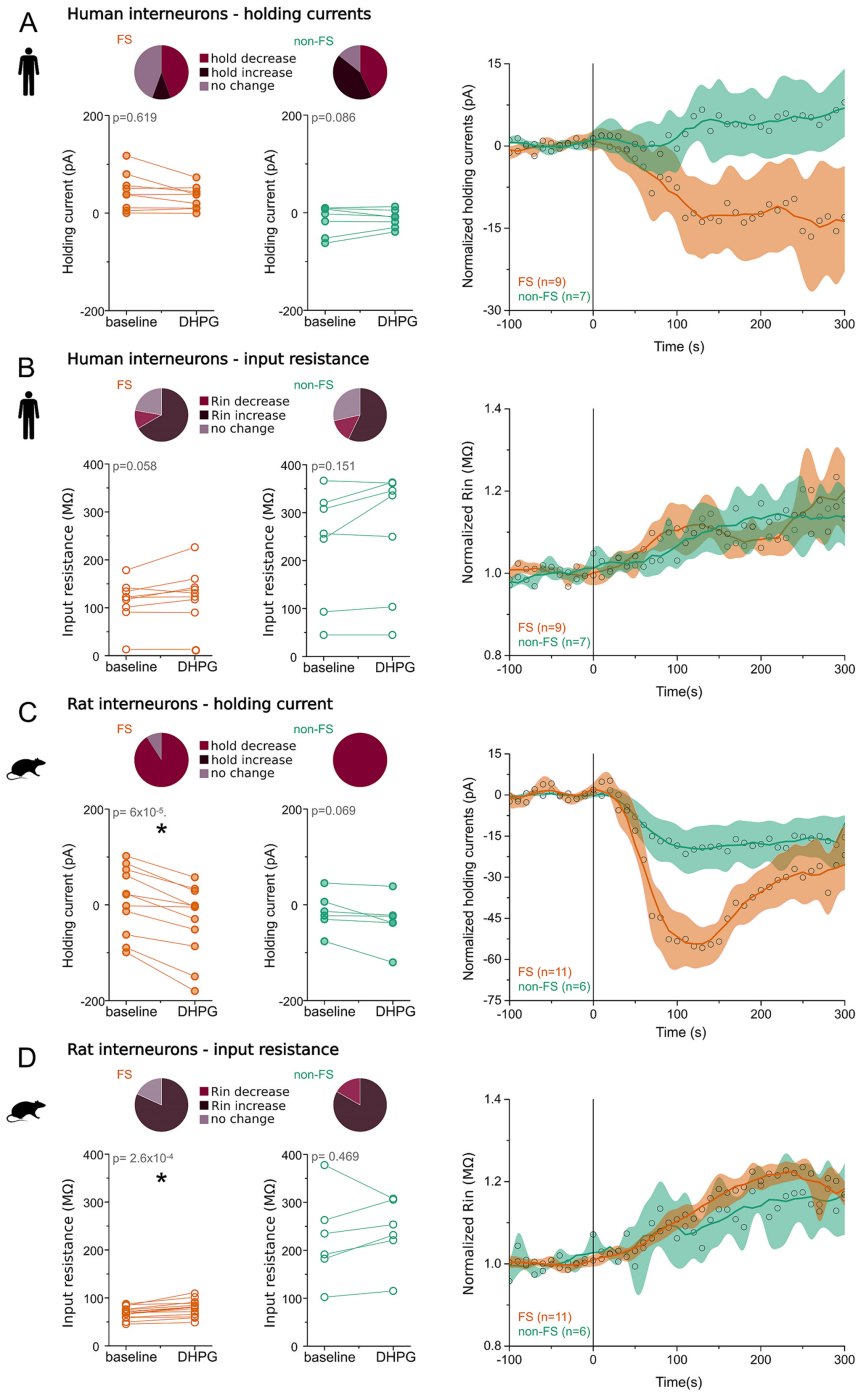
Group I mGluRs induce bidirectional changes in holding current and input resistance in cortical interneurons. **(A)** Human interneurons – holding current. Left: Pie charts show the proportions of interneurons exhibiting a significant decrease (magenta), increase (dark purple), or no significant change (light purple) in holding current following pharmacological activation of mGluR1/5 by DHPG. Line plots display individual data points and population means for fast-spiking (FS, orange) and non-fast-spiking (non-FS, green) interneurons before and after DHPG application. Right: Time course of normalized holding current following DHPG application. Open black circles indicate mean normalized values for FS and non-FS neurons (normalized to baseline). Shaded regions represent the standard error of the mean (SEM; FS: light orange, non-FS: light green). Darker orange and green traces show adjacent-averaged normalized values for FS and non-FS interneurons, respectively. The vertical black line marks the onset of DHPG application. **(B)** Human interneurons – input resistance. Left: Pie charts depict the proportions of cells showing significant decreases (magenta), increases (dark purple), or no change (light purple) in input resistance following mGluR1/5 activation. Line plots show individual and mean Rin values for FS (orange) and non-FS (green) interneurons before and during DHPG application. Right: Time course of normalized input resistance following DHPG application. Open black circles indicate mean normalized Rin values, shaded areas show SEM, and darker traces represent adjacent-averaged data. The vertical black line indicates DHPG onset. **(C)** Same format as in panel **(A)** but for rat interneurons. **(D)** Same format as in panel **(B)** but for rat interneurons.

DHPG induced variable changes in holding current in human interneurons, with heterogeneous responses in both FS and non-FS cells. In non-FS cells, individual experiments showed significant decreases, increases, and no change in holding current in a 3:3:1 ratio, respectively, whereas FS interneurons exhibited a 4:1:4 distribution across these same response categories. At the population level, non-FS interneurons exhibited a trend toward increased holding current following DHPG application (*n* = 7, baseline: −15.45 ± 30.22 pA; DHPG: −12.54 ± 18.39 pA; *p* = 0.086, paired *t*-test), whereas FS interneurons showed a non-significant trend toward decreased holding current (*n* = 9, baseline: 43.91 ± 37.94 pA; DHPG: 31.52 ± 23.69 pA; *p* = 0.619, paired *t*-test; Figure 6A). These group-level differences may be influenced by the more negative baseline holding currents observed in non-FS cells, consistent with their generally more hyperpolarized resting membrane potential.

To assess whether DHPG altered membrane conductance, we examined changes in input resistance (Rin). Across all human interneurons, DHPG induced a significant increase in Rin (*n* = 16, baseline: 165.97 ± 103.24 MΩ; DHPG: 184.14 ± 114.92 MΩ; *p* = 0.015, paired *t*-test), indicating a reduction in membrane conductance. When analyzed by subtype, this increase did not reach statistical significance in FS or non-FS cells considered separately (FS: *n* = 9, baseline: 113.41 ± 45.14 MΩ; DHPG: 126.84 ± 56.70 MΩ; *p* = 0.058; non-FS: *n* = 7, baseline: 233.54 ± 120.25 MΩ; DHPG: 257.81 ± 132.18 MΩ; *p* = 0.151; Figure 6B). At the single-cell level, Rin increased, decreased, or remained unchanged in a 6:1:2 ratio among FS cells and 4:1:2 among non-FS cells, indicating substantial cell-to-cell heterogeneity.

#### 2.3.2 Effects in rodent cortical interneurons

Rodent interneurons displayed a much more uniform response to mGluR activation. In nearly all recorded rodent cells, DHPG application induced a significant decrease in holding current, corresponding to membrane depolarization (*n* = 17, baseline: 0.57 ± 60.93 pA; DHPG: −34.72 ± 66.35 pA; *p* = 0.00002, paired *t*-test). This effect was similarly robust in both FS (*n* = 11, baseline: 9.20 ± 69.97 pA; DHPG: −35.18 ± 75.80 pA; *p* = 0.00006) and non-FS neurons (*n* = 6, baseline: −15.24 ± 40.31 pA; DHPG: −33.89 ± 50.91 pA; *p* = 0.069; Figure 6C).

Consistent with the homogeneity observed in holding current changes, DHPG also elicited consistent changes in Rin in rodent interneurons. At the population level, R_in_ increased significantly following agonist application (all INs: *n* = 17, baseline: 126.26 ± 91.82 MΩ; DHPG: 138.00 ± 86.93 MΩ; *p* = 0.0052). This effect remained significant in FS interneurons when analyzed separately (*n* = 11, baseline: 72.26 ± 10.81 MΩ; DHPG: 83.58 ± 13.96 MΩ; *p* = 0.00026), but not in non-FS cells (*n* = 6, baseline: 225.25 ± 92.60 MΩ; DHPG: 239.12 ± 70.76 MΩ; *p* = 0.469; Figure 6D). The majority of rodent interneurons demonstrated increased Rin following DHPG (FS: 9 of 11 cells; non-FS: 5 of 6), with only one non-FS cell showing a decrease.

### 2.4 Transcriptomic data reveal interneuron subtype–specific expression of group I metabotropic glutamate receptors

Group I metabotropic glutamate receptors, mGluR1 and mGluR5, are widely expressed throughout the brain, but exhibit pronounced regional and cell-type–specific expression patterns (Ferraguti and Shigemoto, 2006; Jian et al., 2010). *GRM1*, which encodes mGluR1, is most highly expressed in the cerebellum and olfactory bulb, whereas *GRM5* expression is more prominent in the neocortex and hippocampus (Romano et al., 1995). Given the heterogeneity of synaptic responses observed in our electrophysiological experiments following group I mGluR activation, we investigated whether these functional differences might arise from interneuron subtype–specific expression profiles.

To this end, we analyzed publicly available single-cell transcriptomic data from the Allen Brain Institute (Tasic et al., 2018; Hodge et al., 2019; Bakken et al., 2021), focusing on layer 2/3 neurons from the human middle temporal gyrus. We compared gene expression in parvalbumin-positive (PV+), vasoactive intestinal peptide–positive (VIP+), and somatostatin-positive (SST+) interneuron populations. PV+ interneurons are typically fast-spiking, whereas VIP+ and SST+ populations include predominantly non-fast-spiking, irregular-spiking, or adapting interneurons (Markram et al., 2004; Tremblay et al., 2016).

**Figure 7:**
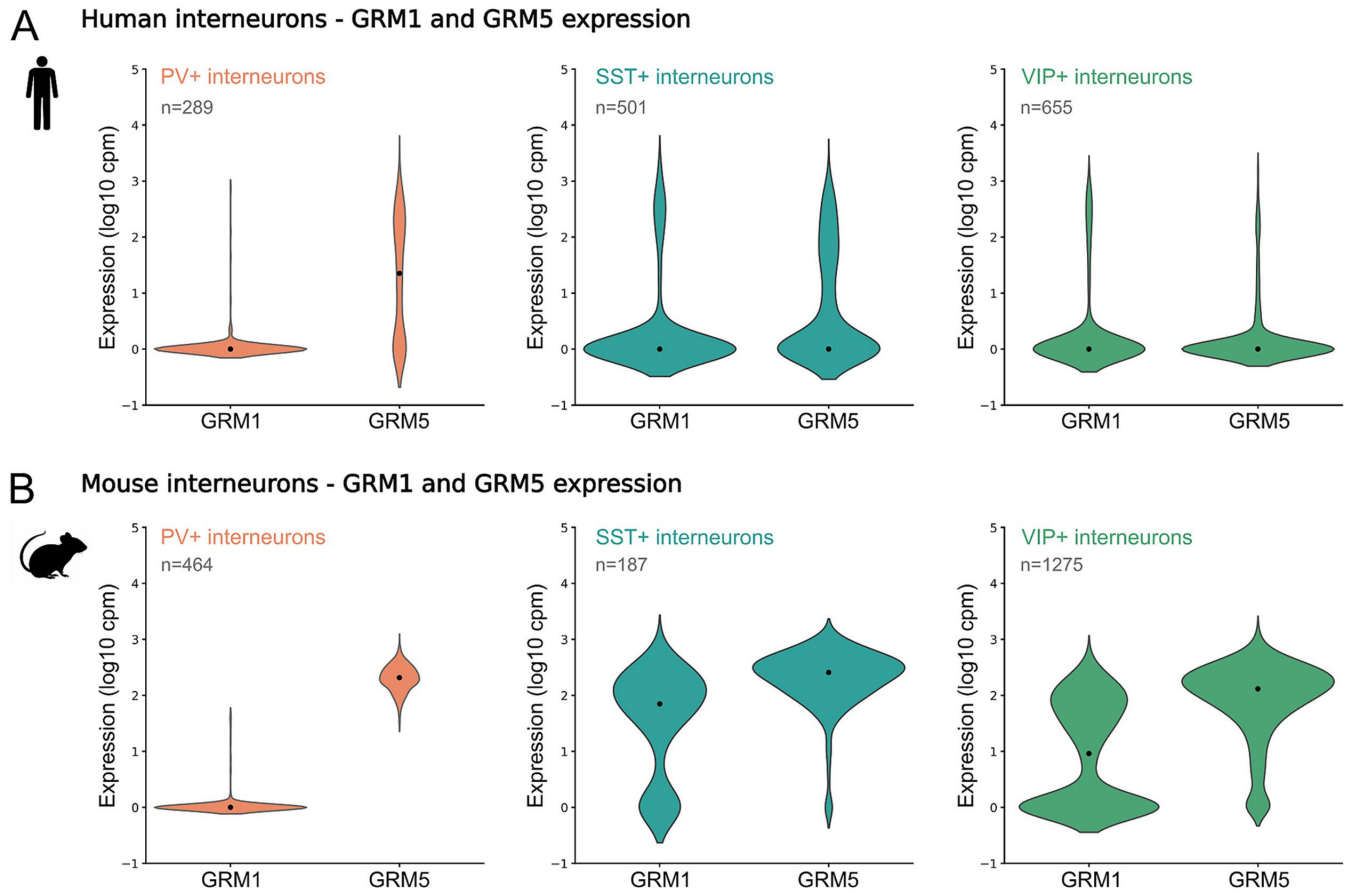
Interneuron subtype–specific expression of group I mGluRs in human middle temporal gyrus. **(A)** Violin plots showing mRNA expression of *GRM1* (mGluR1) and *GRM5* (mGluR5) in PVALB (parvalbumin), SST (somatostatin), and VIP (vasoactive intestinal peptide) positive layer 2/3 human interneurons. Data were obtained from publicly available single-cell RNA-seq datasets from the Allen Brain Institute (https://celltypes.brain-map.org/rnaseq/human/mtg). **(B)** Same as in **(A)** but for mouse neurons (https://celltypes.brain-map.org/rnaseq/mouse/v1-alm).

RNA-seq analysis revealed marked differences in *GRM1* and *GRM5* expression across interneuron subclasses. Among the three major interneuron groups, PV+ (*n* = 289) cells were distinguished by high *GRM5* expression with minimal or undetectable *GRM1* expression. In contrast, SST+ interneurons (*n* = 501) expressed both *GRM1* and *GRM5* at comparable levels, while VIP+ neurons (*n* = 655) exhibited uniformly low expression of both transcripts (Figure 7A). These transcriptomic patterns are consistent with previous reports implicating mGluR5 in synaptic potentiation (Hagena and Manahan-Vaughan, 2022), and align with our electrophysiological finding that a substantial fraction of fast-spiking interneurons in both humans and rodents exhibit synaptic enhancement following group I mGluR activation. Together, these findings support the conclusion that interneuron subtype–specific expression of group I mGluRs contributes, at least in part, to the differential synaptic modulation observed across interneuron classes.

Because comparable single-cell transcriptomic data are not available for our selected rodent model, we next compared the human expression profiles with corresponding datasets from mouse cortex. This analysis revealed a less conserved pattern of group I mGluR expression across species. In mouse interneurons, VIP (*n* = 1,275) and SST (*n* = 187) populations expressed higher levels of *GRM1* and *GRM5* than observed in their human counterparts, whereas PV interneurons (n = 464) exhibited an even more pronounced *GRM5* and *GRM1* ratio (Figure 7B). These interspecies differences suggest that group I mGluR–mediated synaptic modulation may be supported by distinct transcriptional architectures in rodents and humans.

## 3 Discussion

In this study, we characterized the modulatory effects of group I metabotropic glutamate receptors (mGluRs) on excitatory inputs to human cortical interneurons using paired recordings from synaptically connected pyramidal cells and interneurons. We examined excitatory synaptic responses evoked by pyramidal cell action potentials in postsynaptic interneurons and, based on electrophysiological properties, classified interneurons into two major groups: fast-spiking (FS) interneurons, morphologically identified as basket and axo-axonic cells, and a heterogeneous population of non-fast-spiking (non-FS) interneurons. Activation of group I mGluRs with DHPG induced pronounced cell-type and species-specific synaptic modulation. In human FS interneurons, mGluR activation predominantly strengthened excitatory synapses via a mechanism consistent with postsynaptic expression, whereas non-FS interneurons exhibited weaker and more variable effects. In contrast, although rat FS interneurons also showed mGluR-dependent enhancement, the effect was predominantly presynaptic and correlated with baseline release probability. Transcriptomic analyses revealing high *GRM5* and low *GRM1* expression in human parvalbumin-positive (PV+) interneurons further suggest that mGluR5 is the primary mediator of the strengthening of excitatory synapses onto human FS interneurons.

A central finding of this study is the divergence in the mechanisms of type I mGluR-mediated modulation between species. In rat FS interneurons, a trend toward decreased paired-pulse ratio (PPR) and a significant relationship between initial release probability and the magnitude of EPSC amplitude increase suggest a presynaptic mechanism, whereby synapses with low initial release probability are preferentially strengthened. In contrast, human FS interneurons showed no consistent change in PPR and no correlation between baseline release properties and synaptic strengthening, pointing to a postsynaptic locus of expression, potentially reflecting increases in AMPA receptor number or conductance. This mechanistic dissociation highlights an important limitation in translational neuroscience: even when functional outcomes, such as increased synaptic efficacy, are conserved, the underlying cellular mechanisms can differ substantially across species. Consequently, therapeutic approaches targeting presynaptic mechanisms based on rodent models may not generalize directly to the human cortex, emphasizing the importance of human-based experimental validation.

Interneuron-specific synaptic modulation and plasticity are influenced by both functional specialization and structural diversity. Our data, considered alongside previous studies, indicate multiple, context-dependent factors that determine group I mGluR-mediated regulation of synaptic strength in the cortex. While earlier work demonstrated that group I mGluR activation induces long-term depression (LTD) at strong excitatory synapses onto FS interneurons (Le Duigou et al., 2011; Szegedi et al., 2016), we did not observe a relationship between baseline synaptic strength and the magnitude or direction of acute DHPG-induced modulation in human FS interneurons in the present study. In contrast, in our rodent recordings, acute DHPG-induced modulation was consistent with preferential strengthening at synapses with lower initial release probability, supporting a presynaptic contribution to the observed effect. Consistent with our results in Wistar rats, previous reports in rodent cortex showed that synapses with low release probability are more likely to increase their efficacy, whereas strong synapses tend to undergo attenuation of synaptic strength (Chistiakova et al., 2019). These complementary forms of synaptic strength tuning may serve a homeostatic function by narrowing the distribution of synaptic weights to maintain interneurons within an optimal functional range (Bannon et al., 2020). In contrast, our human data add to the growing body of evidence for the diverse roles that group I mGluRs play in regulating cortical circuits and demonstrate acute modulation of synaptic efficacy that is independent of baseline synaptic strength. Multiple factors likely contribute to the diversity of mGluR-mediated modulation in FS interneurons, including synapse-specific receptor composition (van Hooft et al., 2000; López-Bendito et al., 2002; Sun et al., 2009), intracellular calcium signaling dynamics (Goldberg et al., 2003), retrograde signaling mechanisms (Tzounopoulos et al., 2007), postsynaptic activity patterns (Topolnik et al., 2005), and presynaptic release machinery (Reyes et al., 1998; Koester and Johnston, 2005). Accordingly, FS interneurons may express multiple forms of mGluR-dependent plasticity depending on synaptic context (Hainmüller et al., 2014).

The differential effects observed across FS and non-FS interneurons further support the presence of cell-type-specific molecular programs governing group I mGluR signaling. Both electrophysiological classes encompass multiple transcriptomic cell types, likely contributing to the observed heterogeneity. FS interneurons primarily include parvalbumin-positive basket and axo-axonic cells but also incorporate recently identified subtypes such as SST FRZB-expressing cells in the human cortex (Lee et al., 2023). The heterogeneous composition of the non-FS group may further amplify response variability. Consistent with our results, transcriptomic and anatomical evidence from mouse dataset indicates that *GRM5* expression is higher in PV+ neurons than in somatostatin-expressing interneurons (Kerner et al., 1997). While our physiological rodent controls were performed in rats, we utilized the high-resolution mouse transcriptomic atlas for molecular comparisons, assuming a high degree of conservation in interneuron-specific gene expression between these two closely related rodent species. This molecular profile supports the prominent positive DHPG-induced modulation of synaptic transmission observed in FS populations. However, *GRM5* expression is not exclusive to PV+ cells and has also been reported in LAMP5 (neurogliaform) interneurons, which may explain the DHPG-induced EPSC enhancement observed in a subset of non-FS cells, including morphologically identified neurogliaform neurons. Additionally, transcriptomic datasets identify interneurons with little or no detectable *GRM1* or *GRM5* expression, potentially accounting for cases in which mGluR activation failed to alter membrane potential or synaptic strength.

Beyond synaptic modulation, group I mGluR activation consistently altered intrinsic membrane properties in both human and rat interneurons. DHPG application commonly increased input resistance and induced inward currents corresponding to membrane depolarization, consistent with the inhibition of leak potassium conductances such as TASK and TREK channels, a known consequence of G_q_-coupled mGluR1/5 activation (Chemin, 2003). However, the magnitude and polarity of responses varied more widely in human FS interneurons, consistent with the substantial transcriptomic diversity within this population (Chartrand et al., 2023; Lee et al., 2023).

Certain limitations should be considered when interpreting our results. The predominance of small-amplitude excitatory events may have limited our ability to detect LTD at large-amplitude synapses, as described previously (Szegedi et al., 2016). The use of neurosurgical tissue also introduces unavoidable variability related to neocortical area and patient demographic properties, and possible undetected tissue pathology, although resected neocortical tissue was obtained exclusively from non-pathological quasi-control area (Lourenço and Bacci, 2017) which needs to be removed by surgeon to have access to pathological locus in subcortical area. Finally, although EPSC enhancement reversed during washout in some experiments, longer recording durations would be required to conclusively distinguish transient modulation from lasting plasticity.

In conclusion, our study provides direct evidence that group I mGluRs regulate excitatory synaptic transmission in the human cortex in an interneuron-specific manner, with distinct mechanisms operating in FS and non-FS populations. Moreover, the mechanistic divergence between human and rodent FS interneurons highlights the need for caution when extrapolating from animal models to human cortical physiology. These findings advance our understanding of human synaptic regulation and underscore the importance of mGluRs as modulators of cortical inhibition, with implications for neuropsychiatric and neurological disorders characterized by disrupted excitation–inhibition balance.

## 4 Methods

### 4.1 Ethics statement

All procedures were performed according to the Declaration of Helsinki with the approval of the University of Szeged Ethical Committee and Regional Human Investigation Review Board (ref. 75/2014). For all human tissue material written consent was given by the patients prior to surgery. Written informed consent was obtained from all patients before surgery. For underage patients, consent was provided by a parent or legal guardian.

### 4.2 Human cortical tissue

Non-pathological human cortical tissue was obtained from surgically resected brain areas that were removed to treat deep-brain tumors, epilepsy, and hydrocephalus in male and female patients (ages 4–74 years) from the frontal, temporal, and parietal lobes (Supplementary Table 1). The observed mGluR-mediated modulation was not associated with age, sex, cortical region, or clinical indication (Supplementary Figure 1).

Anesthesia was induced with intravenous midazolam and fentanyl (0.03 mg/kg, 1–2 μg/kg, respectively). A bolus dose of propofol (1–2 mg/kg) was administered intravenously. The patients received 0.5 mg/kg rocuronium to facilitate endotracheal intubation. The trachea was intubated, and the patient was ventilated with O_2_/N_2_O mixture at a ratio of 1:2. Anesthesia was maintained with sevoflurane at care volume of 1.2–1.5.

Following surgical removal, the resected tissue blocks were immediately immersed in ice-cold slicing solution and transported from the operating room to the electrophysiology laboratory within 20 min. The solution contained (in mM): 75 sucrose, 84 NaCl, 2.5 KCl, 1 NaH_2_PO_4_, 25 NaHCO_3_, 0.5 CaCl_2_, 4 MgSO_4_, 25 D(+)-glucose, saturated with 95% O_2_ and 5% CO_2_. The solution was maintained on ice in a thermally insulated transport container with continuous oxygenation (95% O_2_ and 5% CO_2_).

### 4.3 Rodent brain tissue

Experiments were performed on Wistar rats (postnatal day 18–30). Animals had *ad libitum* access to standard chow and water. Animals were anesthetized by inhalation of halothane prior to decapitation. Neocortical tissue from the somatosensory cortex was rapidly removed and placed in slicing solution.

### 4.4 Brain slice preparation

From this point slice preparation followed the same protocol for both species. Slices of 320 μm thickness were prepared with a vibrating blade microtome (Microm HM 650 V). The slices were incubated at 36 °C for 1 h, while the slicing solution was gradually replaced by a peristaltic pump (6 mL/min) with a storing solution that contained (in mM) 130 NaCl, 3.5 KCl, 1 NaH_2_PO_4_, 24 NaHCO_3_, 1 CaCl_2_, 3 MgSO_4_, 10 D(+)-glucose, and was saturated with 95% O_2_ and 5% CO_2_.

### 4.5 Electrophysiological recordings

Whole-cell patch-clamp recordings were performed in a submerged recording chamber at 36 °C. Neurons were visualized with infrared differential interference contrast (IR-DIC) microscopy and were patched at depths of 60–130 μm from the slice surface. The recording solution was identical to the storage solution, except that CaCl_2_ was increased to 3 mM and MgSO₄was reduced to 1.5 mM. To avoid EPSCs rundown during longer recordings (Biró et al., 2005; Molnár et al., 2016) micropipettes (5–7 MΩ) were filled with a low [Cl^−^]_i_ solution intracellular solution containing (in mM): 126 potassium-gluconate, 4 KCl, 4 ATP-Mg, 0.3 GaTP-Na_2_, 10 HEPES, 10 phosphocreatine, 10 L-glutamate and 8 biocytin (pH 7.20; 300 mOsm).

Signals acquired with Patchmaster software were low-pass filtered at 8 kHz and digitized at 16 kHz. Presynaptic cells were stimulated using a paired-pulse protocol (PPP) consisting of brief (2–10 ms), paired suprathreshold current injections (800 pA) separated by 60 ms and delivered every 10 s. Baseline recordings were restricted to the first 5 min after the membrane rupture of the interneuron, before DHPG application. Postsynaptic cells were recorded in voltage-clamp mode; holding currents were adjusted slightly when necessary to maintain a stable holding potential. To visualize mean changes across cell groups, holding current values were baseline-subtracted for each cell and then averaged in 10 s time bins. Access resistance was monitored throughout the electrophysiological recordings (25.81 ± 9.38 MΩ), and cells showing a > 25% increase in access resistance were excluded from analysis.

The following pharmacological agents were used: 50 μM (S)-3,5-Dihydroxyphenylglycine (DHPG, Tocris), 25 μM 2-Methyl-6-(phenylethynyl)pyridine hydrochloride (MPEP, Tocris), 10 μM (S)-(+)-*α*-Amino-4-carboxy-2-methylbenzeneacetic acid (LY-367385, Tocris).

### 4.6 Electrophysiological data analysis and statistics

Electrophysiological sub- and suprathreshold features of recorded neurons were measured from voltage responses elicited by 800 ms current steps increasing by 20 pA from −100 pA. Custom-written MatLab (Mathworks) scripts were used to analyze the recorded electrophysiological signals. Resting membrane potential (RMP) was measured immediately after establishing whole-cell configuration with no injected current. The membrane time constant (tau) and input resistance (Rin) were calculated as the averages across all hyperpolarizing current steps, while the sag ratio was measured at the −100 pA step. Rheobase current was defined as the minimal current injection that elicited the first action potential. Action potential (AP) half-width was measured as the time between the rising and decaying phases at half-maximal spike amplitude. Maximum AP upstroke velocity was calculated as the average voltage at the maximum of AP dV/dt over all APs, and minimum AP downstroke velocity as the average voltage at the minimum (absolute maximum) of AP dV/dt over all APs. Normalized interspike interval (ISI) accommodation was computed as the average interspike interval from all sweeps containing at least three APs divided by the number of APs. AP amplitude adaptation was defined as the average change in AP amplitude between consecutive APs.

To identify interneuron subtypes, we generated a 55-parameter feature matrix from the current-step response traces including subthreshold properties, action potential kinetics, and firing pattern adaptations. To account for different units and scales, all features were normalized using a range-scaling method ([0, 1]). Dimensionality reduction was performed using Uniform Manifold Approximation and Projection (UMAP). Based on the local density of our data, the UMAP hyperparameters were set to 4 neighbors and a minimum distance of 0.2, using a Euclidean distance metric. Classification was then achieved by applying K-means clustering (k = 2) directly to the resulting 2D UMAP coordinates. The cluster number was selected *a priori* to distinguish between the two primary physiological classes (Fast-Spiking and Non-Fast-Spiking), and the validity of this partition was subsequently confirmed by silhouette analysis and *post hoc* morphological and physiological verification.

Synaptic responses were analyzed using Fitmaster (HEKA), IgorPro (Wavemetrics) and Origin 7.5 (OriginLab). Synaptic strength and holding current analyses were performed on partially overlapping interneuron samples: EPSC-based synaptic analyses were restricted to recordings with a synaptic connection, whereas holding current changes were quantified in voltage-clamped interneurons regardless of whether a synaptic connection was detected. Evoked EPSC amplitudes were quantified as the difference between the holding current immediately preceding presynaptic stimulation and the peak of the evoked response recorded on the postsynaptic neuron. The significance of DHPG-induced changes in EPSC amplitudes was assessed by comparing ~3 min of baseline recordings with ~3 min of recordings obtained during DHPG application, starting 1 min after wash-in, using the Mann–Whitney test. Synaptic failures (EPSC = 0) were excluded from the analysis. The paired-pulse ratio (PPR) was defined as the second EPSC divided by the first EPSC amplitude evoked by the paired presynaptic action potentials. The latency was calculated as the time interval between the peak amplitude of presynaptic AP and the onset of the EPSP (detected by IgorPro).

All values are reported as mean ± standard deviation (s.d.), unless otherwise specified. Data normality was assessed using the Shapiro–Wilk test, and statistical comparisons were performed using parametric or non-parametric tests, as appropriate and defined for each paradigm. Differences with *p* < 0.05 were considered significant.

### 4.7 Transcriptomic data

Expression of *GRM1* and *GRM5* was quantified using the publicly available single-cell RNA-seq datasets from the Allen Institute for Brain Science Cell Types Database (celltypes.brain-map.org). For human data, we extracted middle temporal gyrus (MTG), layer 2/3 VIP+, PV+ and SST+ interneuron transcriptomes. To make the same comparisons in the mouse, we selected layer 2/3 VIP+, PV+ and SST+ interneurons from the primary visual cortex (VISp) and the anterior lateral motor cortex (ALM).

### 4.8 Histology and reconstruction

Following electrophysiological recordings, slices were immersed in a fixative containing 4% paraformaldehyde, 15% (v/v) saturated picric acid, and 1.25% glutaraldehyde in 0.1 M phosphate buffer (PB; pH = 7.4) at 4 °C for at least 12 h. After several washes with 0.1 M PB, slices were frozen in liquid nitrogen and then thawed in 0.1 M PB, embedded in 10% gelatin, and further sectioned into 60 μm slices. Sections were incubated in a solution of conjugated avidin-biotin-horseradish peroxidase complex (ABC; 1:100; Vector Labs) in Tris-buffered saline (TBS, pH = 7.4) at 4 °C overnight. The enzyme reaction was revealed by the glucose oxidase-DAB-nickel method using 3′3-diaminobenzidine tetra-hydrochloride (0.05%) as chromogen and 0.01% H_2_O_2_ as oxidant. Sections were postfixed with 1% OsO_4_ in 0.1 M PB. After several washes in distilled water, sections were stained in 1% uranyl acetate and dehydrated in an ascending series of ethanol. Sections were infiltrated with epoxy resin (Durcupan) overnight and embedded on glass slides. Perisomatic area of DAB-visualized cells was studied under light microscope for axonal and dendritic morphology. Three-dimensional light-microscopic reconstructions were carried out using a Neurolucida system (MicroBrightField) with a 100 × objective. Reconstructed neurons were quantitatively analyzed with NeuroExplorer software (MicroBrightField).

## Data Availability

The original contributions presented in the study are included in the article/supplementary material, further inquiries can be directed to the corresponding author/s.

## Supplementary material

SUPPLEMENTARY TABLE 1 Patient metadata used in the experiments of this study.

SUPPLEMENTARY FIGURE 1 The magnitude and direction of group I mGluR-mediated modulation of synaptic efficacy is not associated with patient sex, age, cortical region and primary diagnosis. Normalized EPSC change after DHPG application is plotted against patient age. Points correspond to individual synaptic experiments. **(A)** Points are colored by sex. **(B)** Points are colored by region of tissue origin. **(C)** Points are colored by primary diagnosis. **(D)** Points are colored by postsynaptic cell-type.

SUPPLEMENTARY FIGURE 2 The effect of group I mGluR activation on monosynaptic excitation of layer 2/3 non-fast-spiking interneurons in the human cortex. **(A)** Light microscopic reconstruction of the recorded pyramidal cell (dendrites: red; axon: pink) and interneuron (dendrites: blue; axon: light blue). **(B)** Representative firing patterns of the simultaneously recorded presynaptic pyramidal neuron (left) and postsynaptic interneuron (right). **(C)** Action potentials in the presynaptic pyramidal neuron (top) evoked unitary EPSCs in the interneuron (bottom) under voltage-clamp conditions before (left) and after (right) application of the group I mGluR agonist DHPG. **(D)** Time course of EPSC amplitudes and PPR during the experiment. Bath application of DHPG started at time 0 and is indicated by the gray shaded background. **(E)** Left: DHPG induced a significant increase in EPSC amplitudes in this representative experiment (Mann–Whitney test). Boxes represent the mean and interquartile range (IQR) of EPSC amplitudes during baseline and after DHPG application (330–900 s). Asterisks denote statistical significance (***p* < 0.005; ****p* < 0.001).
