# Supplementary material for: Group I metabotropic glutamate receptors differentially modulate excitatory transmission across interneuron types in the human cortex
Source: Front Synaptic Neurosci. 2026 Feb 13;18:1766413. doi: 10.3389/fnsyn.2026.1766413 (PMC12946012; doi:10.3389/fnsyn.2026.1766413)
Supplement: SUPPLEMENTARY TABLE 1 — Patient metadata used in the experiments of this study. [file Table_1.docx]

| Primer diagnosis | Sex | Age | Hemisphere | Region |
| --- | --- | --- | --- | --- |
| hydrocephalus | Female | 73 | right | occipital |
| cavernoma | Male | 70 | left | temporal |
| tumor | Male | 13 | right | frontal |
| shunt | Male | 45 | left | temporal |
| shunt | Female | 70 | left | parietal |
| cyst | Male | 54 | right | frontal |
| tumor | Female | 42 | right | parietal |
| tumor | Female | 11 | right | frontal |
| tumor | Female | 74 | right | frontal |
| hydrocephalus | Female | 21 | right | frontal |
| tumor | Female | 69 | right | temporal |
| tumor | Female | 64 | right | temporal |
| shunt | Female | 65 | left | temporal |
| tumor | Male | 4 | left | temporal |
| shunt / malformation | Male | 11 | left | temporal |
| shunt | Male | 72 | right | temporal |
| tumor | Male | 47 | right | temporal |
| tumor | Male | 67 | right | temporal |
| hydrocephalus | Female | 74 | right | temporal |
| shunt | Female | 61 | right | temporal |
| tumor | Female | 62 | left | temporal |
| tumor | Male | 53 | right | temporal |
| tumor | Female | 45 | right | frontal |
| tumor | Male | 38 | right | Temporal |
| tumor | Male | 73 | male | Frontal |
| tumor | Male | 76 | right | Temporal |
| shunt | Male | 19 | right | Parietal |
| colloid cyst | Female | 13 | right | Frontal |
| tumor | Male | 65 | right | Temporal |
| tumor | Male | 13 | right | Frontal |
| tumor | Female | 56 | left | Temporal |
| aneurysm | Male | 40 | right | Temporal |
| tumor | Male | 58 | left | Temporal |
| tumor | Male | 65 | right | Temporal |
| shunt | Female | 50 | right | Parietooccipital |
| tumor | Male | 65 | left | Ventral |
| malformation | Male | 34 | left | Cerebellar |
| tumor | Female | 63 | right | Temporal |
| hydrocephalus | Female | 70 | right | Parietal |
