# Supplementary figures and images for: Group I metabotropic glutamate receptors differentially modulate excitatory transmission across interneuron types in the human cortex
Source: Front Synaptic Neurosci. 2026 Feb 13;18:1766413. doi: 10.3389/fnsyn.2026.1766413 (PMC12946012; doi:10.3389/fnsyn.2026.1766413)

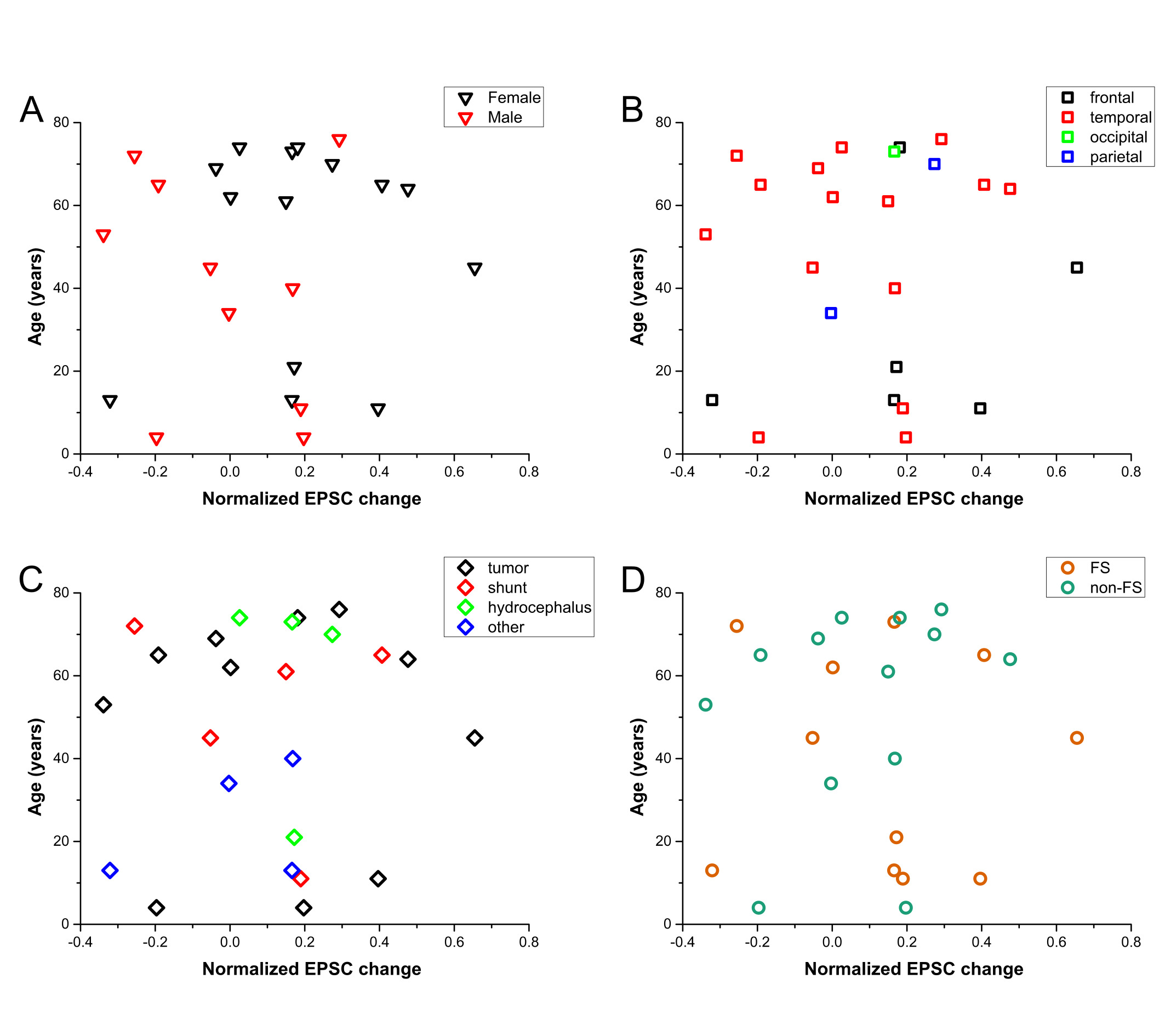

Supplement: SUPPLEMENTARY FIGURE 1 — The magnitude and direction of group I mGluR-mediated modulation of synaptic efficacy is not associated with patient sex, age, cortical region and primary diagnosis. Normalized EPSC change after DHPG application is plotted against patient age. Points correspond to individual synaptic experiments. (A) Points are colored by sex. (B) Points are colored by region of tissue origin. (C) Points are colored by primary diagnosis. (D) Points are colored by postsynaptic cell-type. [file Image_1.jpeg]

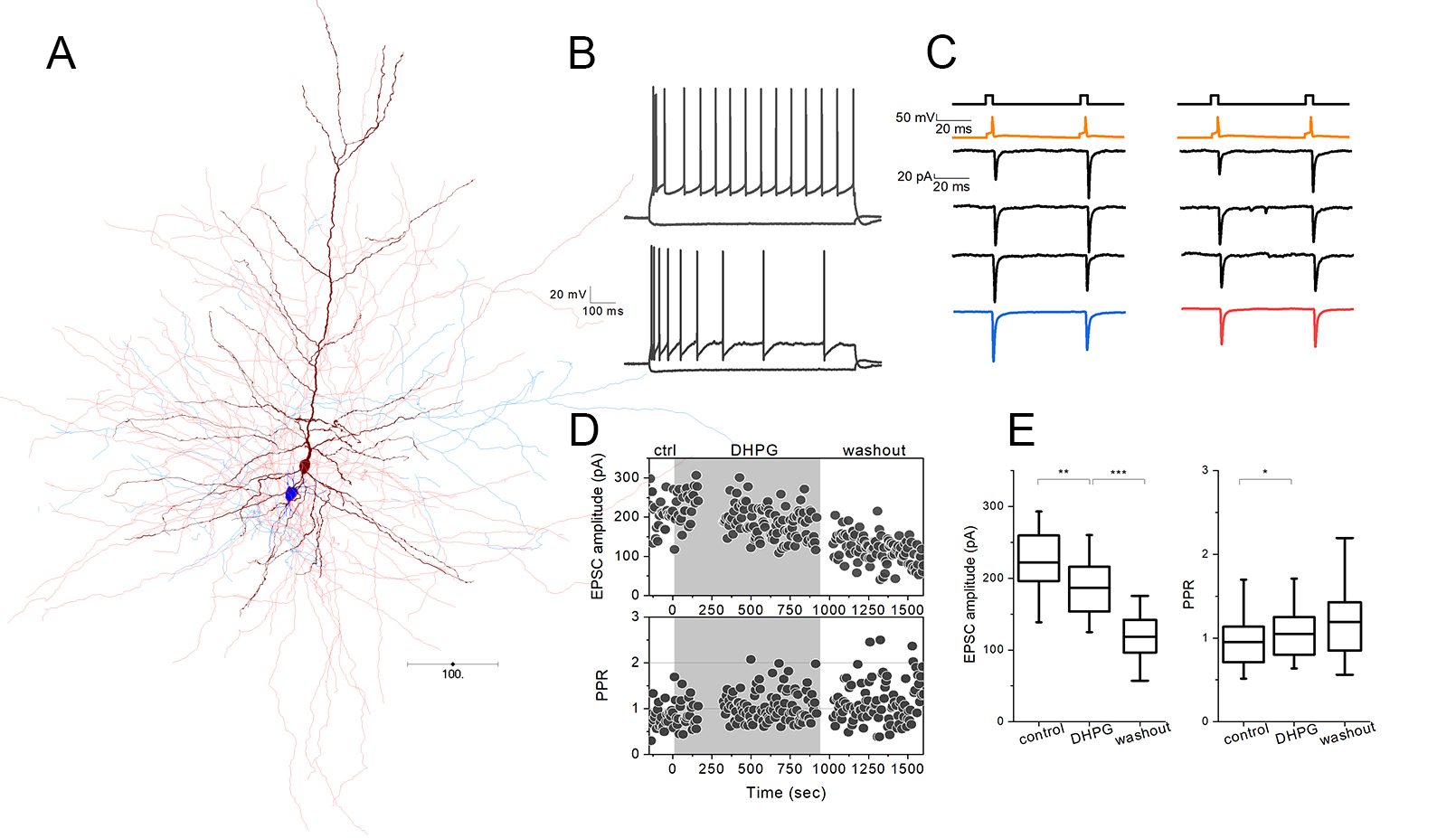

Supplement: SUPPLEMENTARY FIGURE 2 — The effect of group I mGluR activation on monosynaptic excitation of layer 2/3 non-fast-spiking interneurons in the human cortex. (A) Light microscopic reconstruction of the recorded pyramidal cell (dendrites: red; axon: pink) and interneuron (dendrites: blue; axon: light blue). (B) Representative firing patterns of the simultaneously recorded presynaptic pyramidal neuron (left) and postsynaptic interneuron (right). (C) Action potentials in the presynaptic pyramidal neuron (top) evoked unitary EPSCs in the interneuron (bottom) under voltage-clamp conditions before (left) and after (right) application of the group I mGluR agonist DHPG. (D) Time course of EPSC amplitudes and PPR during the experiment. Bath application of DHPG started at time 0 and is indicated by the gray shaded background. (E) Left: DHPG induced a significant increase in EPSC amplitudes in this representative experiment (Mann–Whitney test). Boxes represent the mean and interquartile range (IQR) of EPSC amplitudes during baseline and after DHPG application (330–900 s). Asterisks denote statistical significance (**p < 0.005; ***p < 0.001). [file Image_2.jpeg]
